# Preparation of gemini surfactant/graphene oxide composites and their superior performance for Congo red adsorption†

**DOI:** 10.1039/c8ra10025j

**Published:** 2019-02-08

**Authors:** Shuai He, Xingli Liu, Ping Yan, Anqi Wang, Jinzhu Su, Xin Su

**Affiliations:** College of Chemistry and Environmental Protection Engineering, Southwest Minzu University Chengdu 610041 China heshuaifish@126.com +86-028-85928021; Polymer Research Institute, State Key Laboratory of Polymer Materials Engineering, Sichuan University Chengdu 610065 China xinsu@scu.edu.cn +86-028-85461786

## Abstract

Gemini surfactant/GO composites (10-2-10/GO, 12-2-12/GO, and 14-2-14/GO) have been successfully prepared using three gemini surfactants with different tail chain lengths. The morphology and physicochemical properties of the as-synthesized composites were characterized by scanning electron microscopy, transmission electron microscopy, Fourier transform-infrared spectroscopy, X-ray diffraction, and X-ray photoelectron spectroscopy. The gemini surfactant/GO composites were applied to the adsorption of Congo red dye, and from the experimental data, optimum adsorption conditions, adsorption kinetics, and isotherms were obtained. The removal process was favorable at acidic pH and reached equilibrium in ∼60 min. The results showed that the pseudo-second-order model and the Langmuir adsorption isotherm were a good fit for the adsorption of Congo red onto gemini surfactant/GO composites. Compared with other adsorbents reported in the literature, these composites showed superior Congo red adsorption capabilities, with absorption capacities as high as 2116, 2193, and 2325 mg g^−1^ for 10-2-10/GO, 12-2-12/GO, and 14-2-14/GO, respectively. Moreover, the adsorption capacities were more than 1000 mg g^−1^ even for the fifth cycle. The results of the present study substantiate that the gemini surfactant/GO composites are promising adsorbents for the removal of organic dyes in wastewater treatment.

## Abstract 2

Gemini surfactant/GO composites were prepared for the removal of Congo red dye, and show excellent adsorption capacities and reusabilities.

## Graphical abstract

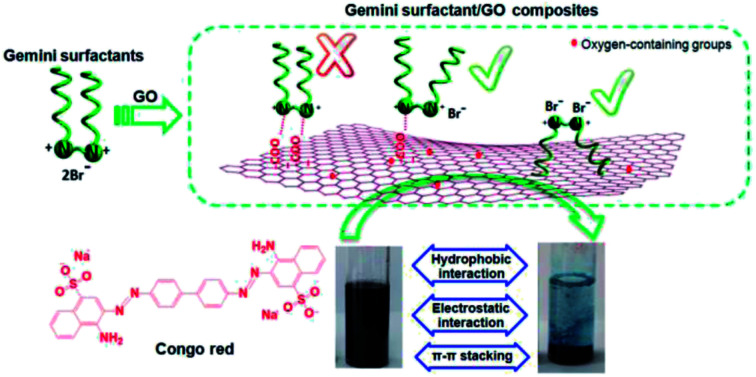

## 1. Introduction

Global water resources are suffering from escalating serious pollution. The United Nations reports state that 300–500 million tonnes of contaminants are discharged into river streams annually, and this phenomenon may cause water shortage to one-third of the world's population by 2030.^1^ Water pollution is caused by numerous factors, such as heavy metal ions, dyes, and other contaminants.^2–4^ Dyes are considered as the primary contaminant of wastewater because of their widespread use in industry.^5^ Studies have reported that many dyes, such as Congo red, rhodamine B, and methyl blue, may have carcinogenic and mutagenic effects on animals and humans.^6^ Therefore, dye concentrations in industrial waste effluents should be strictly limited before emission to the environment.

Different physicochemical methods, for example, adsorption, coagulation/flocculation, catalytic ozonation, and electrocatalytic degradation, have been applied to eliminate dyes from aqueous matrices.^7–10^ Among these methods, adsorption is the most advantageous water treatment technique because of its low process cost, simple operation, minimal sludge production, high efficiency, and reusability.^11–13^ Various materials have been used to adsorb organic dyes, such as clays, mesoporous gels, organic–inorganic hybrids, magnetic particles, activated carbon, and graphene oxide (GO).^14–19^ As a new promising material, GO is one of the most preferred adsorbents because of the unique layered structure, large surface area, and high adsorption capabilities of this material. However, stable dispersed GO is difficult to separate from the adsorption system, leading to additional steps needed for collection after adsorption and the loss of the adsorbent. Moreover, the risk of GO as a nanomaterial to aquatic environment should not be ignored.^20^ These problems limit the industrial application of GO adsorbent.

These drawbacks are overcomed by combining GO with various materials to form composite precipitation.^21^ Notably, GO-based composites modified by surfactants have recently attracted significant attention because of the easy availability and high efficiency of surfactants.^22–24^ Liang *et al.*^25^ reported for the first time that cationic surfactants were used to modify GO by ionic interactions, thereby reducing the hydrophilicity of the graphene composites. Subsequently, Yusuf and coworkers prepared a graphene composite intercalated with cetyltrimethylammonium bromide (CTAB), and the obtained composite showed great removal capacity for organic dyes and good recovery.^23^ Recently, Mahmoodi *et al.*^26^ and our group^27^ also found that CTAB could efficiently enhance graphene removal of organic dyes because of synergistic effect of electrostatic attraction and hydrophobic interaction. Generally, the positively charged surfactants can be anchored on negatively charged carboxyl group of GO by electrostatic attraction. Meanwhile, the hydrophobic tail chain of surfactants enhances the hydrophobicity of products. These two factors determine the surface charge and hydrophobicity of graphene and markedly influence the adsorption performance and the recyclability. Therefore, the choice of suitable surfactants is critical to the industrial application of GO-based adsorbents.

Gemini surfactants, with two quaternary ammonium head groups linked by a spacer and with each headgroup attached to one hydrophobic tail, can provide improved excellent chemical stability and strong adsorption for various pollutants.^28^ Such unique properties may furnish gemini surfactants to fabricate novel GO-based composites. On the one hand, cationic gemini surfactants have two cationic headgroups, which may be adsorbed onto negatively charged carboxyl groups of GO to form composites. On the other hand, the double hydrophobic tails of surfactants would greatly enhance the hydrophobicity of composites, resulting in the efficient separation and quick recovery of the absorbents.

In this study, we prepared for the first time a series of gemini surfactant/GO composites by a simple mixing process using gemini surfactants with different tail chain lengths. The morphology and physicochemical properties of the as-obtained composites were characterized by scanning electron microscopy (SEM), transmission electron microscopy (TEM), Fourier transform-infrared (FT-IR) spectroscopy, X-ray diffraction (XRD), and X-ray photoelectron spectroscopy (XPS). Adsorption properties of Congo red dye onto gemini surfactant/GO composites were evaluated by studying the effects of various factors, namely, pH, contact time, initial solution concentration, and tail chain length of surfactants. All three gemini surfactant/GO composites presented excellent adsorption capacities, which were comparable with other high-performance absorbent materials. In addition, the adsorption kinetics, isotherms, and reusability of these adsorbents were also systematically analyzed.

## 2. Materials and methods

### 2.1 Materials

Three kinds of gemini surfactants with different chain lengths (C_10_H_21_N^+^(CH_3_)_2_–(CH_2_)_2_–N^+^(CH_3_)_2_C_10_H_21_·2Br^−^, C_12_H_25_N^+^(CH_3_)_2_–(CH_2_)_2_–N^+^(CH_3_)_2_C_12_H_25_·2Br^−^ and C_14_H_29_N^+^(CH_3_)_2_–(CH_2_)_2_–N^+^(CH_3_)_2_C_14_H_29_·2Br^−^, abbreviated as 10-2-10, 12-2-12 and 14-2-14, respectively) were kindly provided by Chengdu Institute of Organic Chemistry, China. Congo red (C_32_H_22_N_6_O_6_S_2_Na_2_) and graphite were obtained from Aladdin, China. Unless otherwise noted, all other chemicals were reagent grade. The water used for experiments was prepared with deionized water (DI water).

### 2.2 Synthesis of gemini surfactant/GO composites

These composites can be obtained by simple mixing of GO and gemini surfactants. GO was synthesized from crystalline flake graphite by the modified Hummers method.^29^ A gemini surfactant/GO composite was prepared as follows: a 100 mL of GO dispersion (2 mg mL^−1^) was mixed with 100 mL gemini surfactant aqueous solution (20 mg mL^−1^). After being vigorously stirred for one hour, the brown precipitation was obtained. The resulting precipitation was filtered, then sequentially washed with DI water. It was tested by adding silver nitrate, and no precipitation indicated the cleanup of impurities. Lastly, a brown gemini surfactant/GO composite was obtained by vacuum freeze-drying. With such a process, a series of composites were prepared with different gemini surfactants, and the corresponding composites were denoted as 10-2-10/GO, 12-2-12/GO and 14-2-14/GO, respectively.

### 2.3 Characterization and instruments

Static contact angle was tested by depositing water on composite surface and then the data was analyzed by SDC-100S software. The FT-IR spectra were recorded using a Nicolet MX-1E FTIR spectrophotometer, operating in a spectral range of 4000–400 cm^−1^. SEM observation was carried out by JEOL JSM-7500F with an accelerating voltage of 2 kV. TEM images were obtained using a Hitachi H-600 microscope. XRD patterns of samples were performed by a Rigaku DMAX2200 with Ni-filtered Cu Kα radiation over a scanning range of 5° to 60° at an X-ray power of 36 kV and 20 mA. XPS experiments were measured by a Kratos XSAM800 XPS. The zeta-potential values of samples were tested by Malvern Zetasizer Nano-ZS.

### 2.4 Adsorption and regeneration procedure

The adsorption process was carried out at 298 K in aqueous solution environment. Typically, Congo red solution and composite were added in glass tubes and the mixture was shaken at 300 rpm. The desired pH of solution was adjusted by adding 0.1 mol L^−1^ of HCl or NaOH before the adsorption experiment. According to previous literatures, the residual Congo red concentration at different time intervals was determined using a UV-Vis spectrophotometer (Mapada Co., Ltd, China) at 498 nm.^30–32^ The influence of pH value, Congo red concentration and adsorption time were studied. The equilibrium adsorption amounts of composites were obtained using the following mass balance relationship:

1 *Q*_e_ = (*C*_0_ − *C*_e_)*V*/*m*

where *Q*_e_ (mg g^−1^) is the amount of dye adsorbed into composites; *C*_0_ and *C*_e_ (mg L^−1^) are the initial and equilibrium concentrations of dye in the solution, respectively; *V* (L) is the solution volume; and *m* (g) is the mass of composites used.

After adsorption, the dispersion system was centrifuged at 3000 rpm for 3 min and 10 mL ethanol was added in four portions to remove Congo red adsorbed on composites. Next, the adsorbent was dried for reuse.

## 3. Results and discussion

### 3.1 Preparation and characterization of gemini surfactant/GO composites

**Fig. 1:**
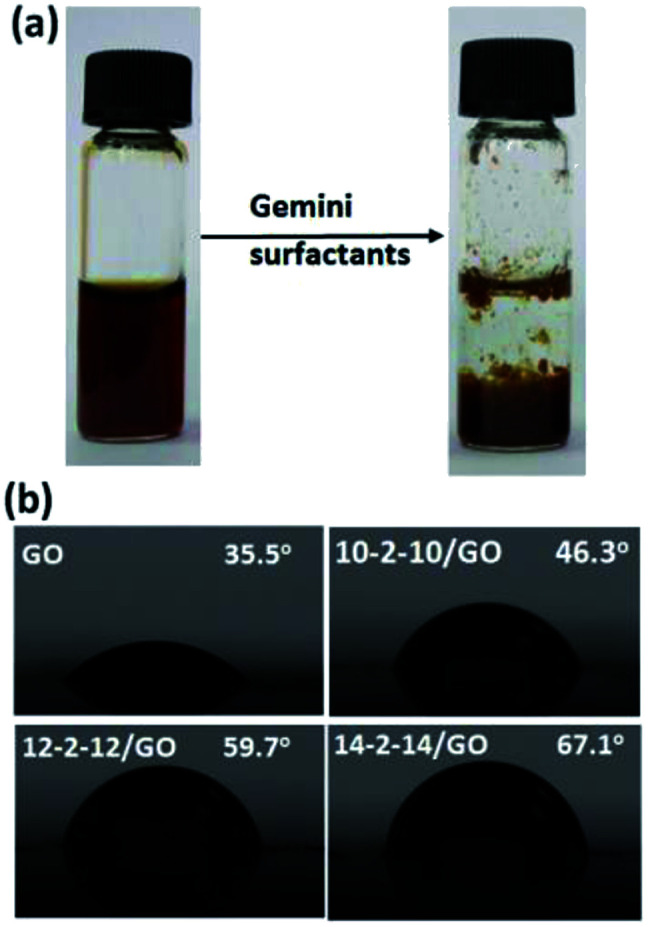
(a) Photographs of the GO solution before and after adding gemini surfactants, (b) static contract angles of GO and gemini surfactant/GO composites.

The gemini surfactant/GO composites were prepared by directly blending GO solution with corresponding gemini surfactant solutions. The resulting solution immediately precipitated, and composites formed (Fig. 1a). The colorless upper layer solution contained no GO residue. Hence, GO completely reacted, and the entire composites remained in the precipitates. These composites could be easily separated by centrifugation at 3000 rpm for 3 min. The hydrophilic/hydrophobic property of the composites was evaluated by static contact angle in Fig. 1b. GO was relatively hydrophilic and showed a contact angle of ∼35.5°. However, the contact angles of 10-2-12/GO, 12-2-12/GO and 14-2-14/GO composites were 46.3°, 59.7° and 67.1°, and these values indicate that these materials were considerably more hydrophobic than GO. Several studies have found that the adsorbed surfactants had their hydrocarbon chain toward the liquid phase and exhibited an increase in hydrophobicity of the material surface.^33,34^ In our experiments, the two hydrocarbon chains of the gemini surfactant molecule may be toward the water phase, and this characteristic enhanced the hydrophobicity of the as-obtained composites. The difference of contact angle of the composites was due to the different length of tail chain of surfactants, and the surfactants with longer tail chain caused the stronger hydrophobicity.^35^ The more detailed mechanisms will be discussed by subsequent characterizations.

**Fig. 2:**
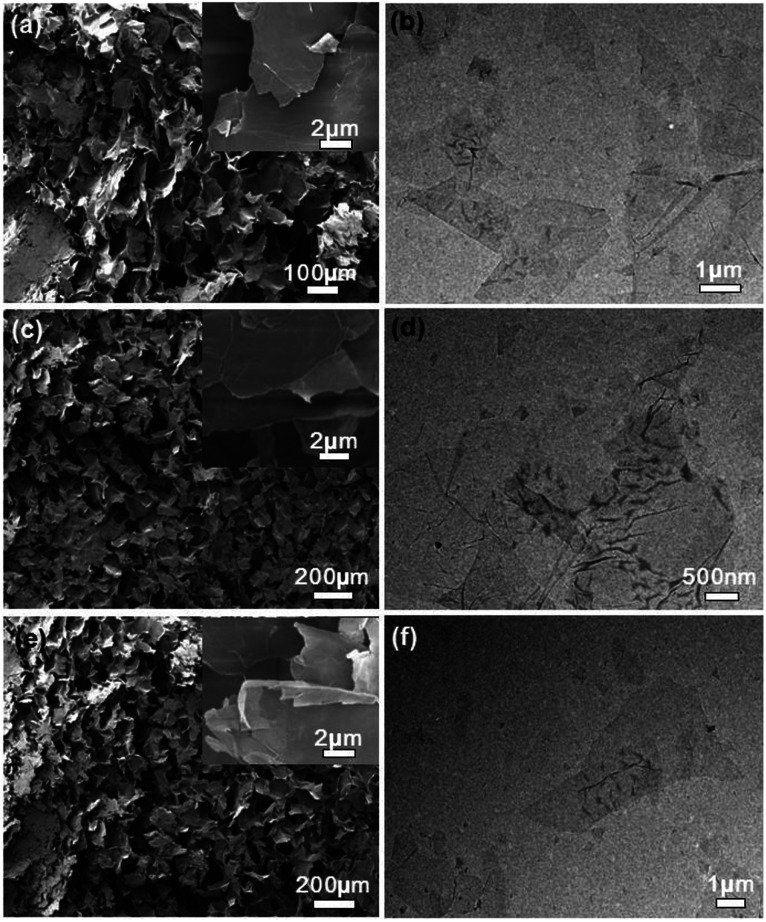
SEM and TEM images of gemini surfactant/GO composites: (a and b) 10-2-10/GO, (c and d) 12-2-12/GO, and (e and f) 14-2-14/GO. Insets of (a, c and e) show the magnified view of the layered graphene.

The SEM and TEM images of the gemini surfactant/GO composites are shown in Fig. 2. All samples exhibited irregular and loose frameworks of large sheets in SEM images. Few-layered sheets with several wrinkles were found in TEM images and higher magnification SEM images. This result suggested that the characteristic layered structure of graphene nanosheets was retained in these composites. Furthermore, no obvious surfactant aggregation was found in the sheet interface. It indicated the uniform distribution of modifiers on graphene surface. Layered structure could enhance the surface area of composites, and this characteristic was crucial for further application in dye adsorption.

**Fig. 3:**
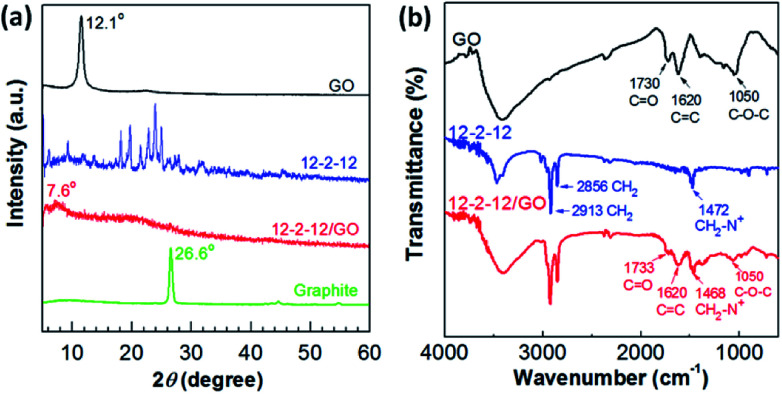
(a) XRD patterns and (b) FT-IR spectra of GO, 12-2-12, and 12-2-12/GO composite.

The as-obtained composites were analyzed by XRD and FT-IR spectroscopy to further study the mechanism of the gemini surfactant adsorbed on GO. The typical XRD patterns of GO, 12-2-12, and 12-2-12/GO composite are shown in Fig. 3a. The (002) peak of GO occurred at 12.1° and corresponded to an interlayer distance of ∼0.810 nm, as determined by Bragg's law. Meanwhile, the 12-2-12 composite showed many diffraction peaks because of the crystallization of quaternary ammonium salts. However, only one diffraction peak at 7.6° appeared in the XRD pattern of the 12-2-12/GO composite. This peak corresponded to an interlayer distance of 1.284 nm (bigger than that of GO). This phenomenon indicated that the product was not a simple mixture of 12-2-12 and GO, and the new composite was formed through some kind of attraction force between the gemini surfactants and GO. Additionally, no graphite peaks appeared in XRD patterns of composites, indicative of the good exfoliating effect.^36^Fig. 3b displays the FT-IR spectra of GO, 12-2-12, and 12-2-12/GO composite. The FT-IR peaks of GO corresponded to the oxygen functionalities, namely, C–O–C (*ν*_C–O_ at 1050 cm^−1^), C=C (*ν*_C=C_ at 1620 cm^−1^), and C–O in carboxylic acid moieties (*ν*_C=O_ at 1730 cm^−1^). Although these oxygen-containing functional groups provided GO with good dispersibility in water, the difficult collection after adsorption had to be performed. After modification by the gemini surfactant, the oxygen-containing groups on original GO were retained in the composite, such as the negatively charged carboxylic acid (*ν*_C=O_ at 1733 cm^−1^). Moreover, the typical surfactant absorption-related features were shown in the FT-IR spectrum of the 12-2-12/GO composite. These peaks at 2913 cm^−1^, 2856 cm^−1^, and 1468 cm^−1^ were assigned to the stretching modes of CH_2_ in the hydrocarbon chains and the symmetric deformation modes of CH_3_–N^+^ in the cationic headgroups, respectively.^37^ Similar to most cationic surfactants, the quaternary ammonium group in the gemini surfactants was assumed to have the ability to bind with the carboxylic acid group on GO *via* electrostatic interaction.^38–40^ Hence, the results confirmed the successful preparation of the gemini surfactant/GO composite.

**Fig. 4:**
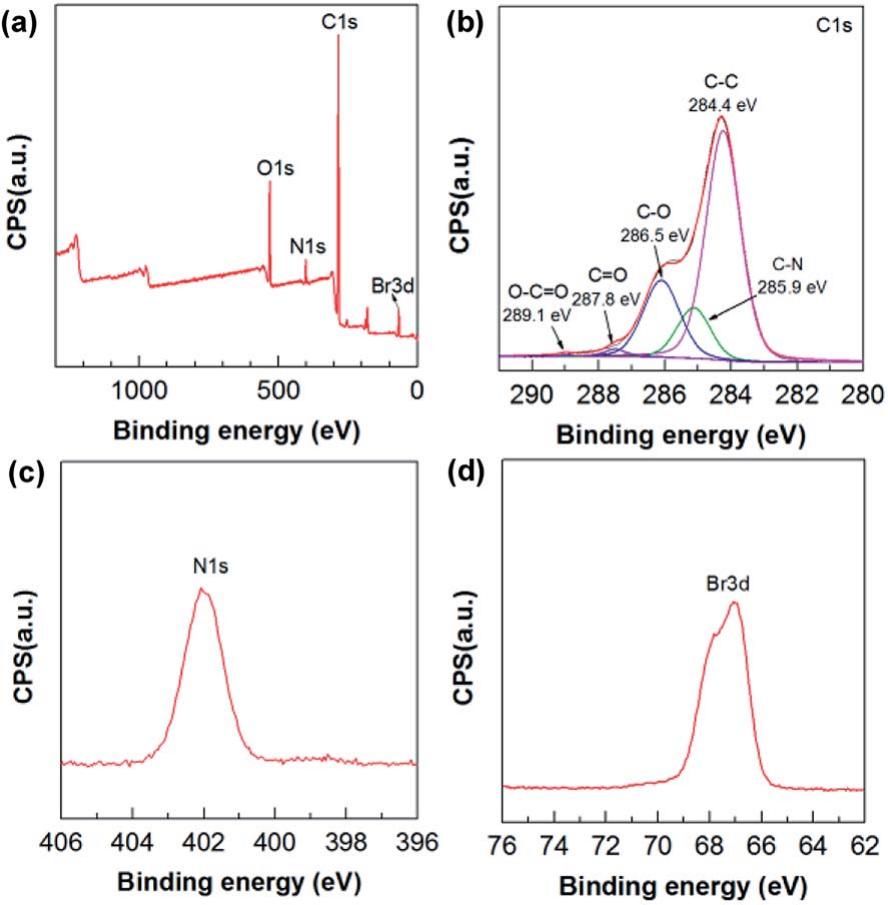
(a) XPS survey spectrum, (b) C 1s XPS spectrum, (c) N 1s XPS spectrum and (d) Br 3d XPS spectrum of 12-2-12/GO composite.

The chemical composition of the composites was also characterized using XPS. C (82.45 at%), O (11.79 at%), N (3.38 at%), and Br (2.38 at%) were detected in the 12-2-12/GO composite (Fig. 4a). The C/O ratio (∼6.99) of 12-2-12/GO composite was higher than that of GO sample (∼2.48) because of the large amount of –CH_2_– in the surfactants. Five types of carbon bonds were observed in the C 1s XPS spectrum as follows (Fig. 4b): C–C (284.4 eV), C–N (285.9 eV), C–O (286.5 eV), C=O (287.8 eV), and O–C=O (289.1 eV). The N (402.3 eV) was derived from quaternary ammonium of the surfactants, and the Br (402.2 eV) incorporated into the composites was confirmed by the Br 3d XPS spectrum (Fig. 4d). The N and Br elements for 10-2-10/GO and 14-2-14/GO are also shown in Table S1 in ESI.† These data proved that the gemini surfactants had been successfully integrated into the composites.

**Fig. 5:**
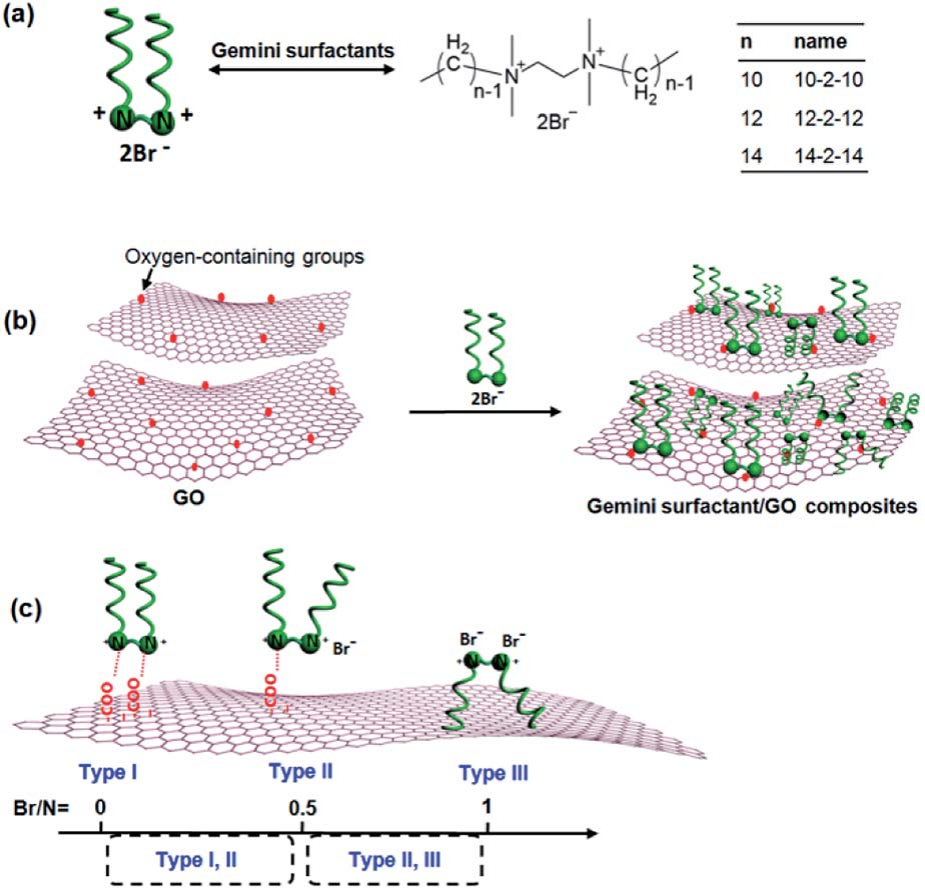
(a) Chemical structures of the gemini surfactants. (b) Illustration of the preparation of the gemini surfactant/GO composites. (c) Three types of gemini surfactant adsorbed on GO.

A series of gemini surfactant/GO composites (10-2-10/GO, 12-2-12/GO and 14-2-14/GO) were prepared by a simple mixing process (Fig. 5a and b). While GO and the gemini surfactant solutions were mixed, two mechanisms may be responsible for the formation of the composites. On the one hand, the positively charged headgroup of gemini surfactants could adsorb on the negatively charged carboxyl of GO by electrostatic interaction. On the other hand, the hydrophobic alkyl chain of gemini surfactants might anchor on hydrophobic region of GO by hydrophobic binding force. Therefore, three possible types of gemini surfactant on graphene surface were speculated as follows (Fig. 5c): (I) both two cationic headgroups of gemini surfactant molecule were adsorbed onto the negatively charged surface of GO by electrostatic interaction, and the Br^−^ of surfactants should not been observed in the gemini surfactant/GO composites; (II) only one headgroup of surfactant molecule was anchored on GO *via* electrostatic interaction, and the atom mole percentage ratio (Br/N) of Br to N in composites was 0.5; and the (III) surfactant molecule was integrated *via* hydrophobic binding force, and the Br/N ratio was 1. Table 1 presents that the Br/N ratios of the gemini surfactant composites were calculated to be *ca.* 0.53, 0.70, and 0.63 for 10-2-10/GO, 12-2-12/GO, and 14-2-14/GO, respectively. All Br/N ratios were between 0.5 (Type II) and 1 (Type III), indicating that Types II and III existed in the composites. Hence, the composites were formed by the coexistence of hydrophobic binding and electrostatic interactions. Given that the carboxyl groups are generated randomly on the surface of graphene, the distance between two adjacent carboxyl groups cannot be exactly equal to that between two headgroups of the gemini surfactant molecule. Thus, Type I was nonexistent.

**Table 1:** Maximum adsorption capacities (*Q*_m_), balance times, and solution pH of various adsorbents towards Congo red

Type of adsorbent	*Q*_m_ (mg g^−1^)	Time (min)	pH	Reference
GO	150	3000	7	28
GO/chitosan/silica fibers	294	900	3	21
Hierarchical hollow γ-Al_2_O_3_	417	2	6.8	50
γ-Al_2_O_3_	466	100	6.5	54
Polysaccharide/GO	789	30	3	41
MIL-68 (In) nanorods	1204	60	2	51
α-Fe/Fe_3_O_4_ composite	1297	3	7	52
Porous hierarchical MgO	2409	60	7	53
CTAB/GO composite	2767	60	3	27
10-2-10/GO composite	2116	60	3	Present study
12-2-12/GO composite	2193	60	3	Present study
14-2-14/GO composite	2325	60	3	Present study

### 3.2 Adsorption of Congo red by the gemini surfactant/GO composites

The introduction of gemini surfactants will greatly change the surface properties of GO, such as the adsorption properties for dye pollutants. The composites possess good stability in water (Fig. S1†). It was demonstrated using Congo red as the model pollutant to research the removal process of pollutants.

#### 3.2.1 Effect of initial pH value on adsorption

**Fig. 6:**
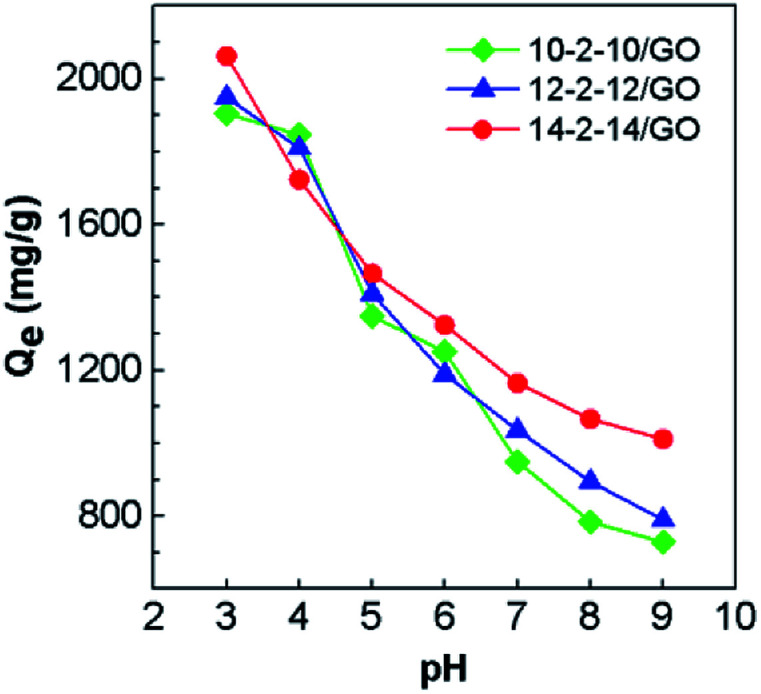
Effect of pH values on Congo red adsorption for the gemini surfactant/GO composites (adsorbent dose: 5 mg, Congo red concentration: 500 mg L^−1^ and volume: 25 mL).

The alteration of pH values can change the form of the pollutant and the surface charge of composites. Thus, pH values might have a significant effect on the adsorption of the composites. Fig. 6 displays the effect of pH values on adsorption of Congo red onto composites. The adsorption capacities for the gemini surfactant/GO composites decreased with increasing pH from 3 to 9. As shown in Fig. S2,† Congo red does not precipitate even under acidic conditions. The highest adsorption capacity was achieved at pH 3. For example, the highest *Q*_e_ for 10-2-10/GO, 12-2-12/GO, and 14-2-14/GO were 1905, 1949, and 2063 mg g^−1^, respectively. The outstanding adsorption performance could be attributed to the different interactions between the gemini surfactant/GO composites and Congo red in terms of surface charge and chemical structure. After the gemini surfactant modification, the zeta potential of the complex increased to 49.28 ± 3.21 mV (12-2-12/GO at pH ≈ 7), markedly higher than that of the initial GO (−42.35 ± 2.79 mV). Congo red is an anionic dye, which could be adsorbed on the surface of positively charged composites. Therefore, the high adsorption capacities for Congo red can be obtained. Moreover, another possible reason for the high adsorption is the strong π–π stacking interactions between aromatic structure of Congo red and hexagonal arrays of the carbon atoms in graphene.^41,42^ The follow-up experiments were performed at pH 3 unless otherwise noted, because the removal process was favorable at low pH.

#### 3.2.2 Effect of gemini surfactants on the adsorption

**Fig. 7:**
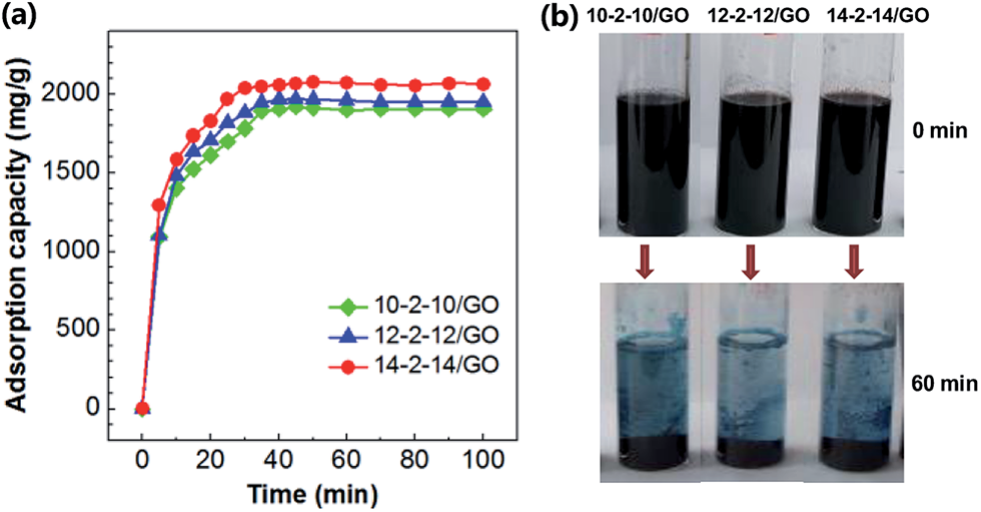
(a) Time-dependent adsorption of Congo red onto the gemini surfactant/GO composites (adsorbent dose: 5 mg, pH: 3, Congo red concentration: 500 mg L^−1^ and volume: 25 mL). (b) Photograph of Congo red aqueous solution treated by composites at 0 min and 60 min.

The Congo red adsorption capacities on the gemini surfactant/GO composites are shown in Fig. 7a, while Fig. 7b presents the photograph of Congo red aqueous solution treated by composites. Congo red was a pH indicator, which showed characteristic blue in acidic environment at 0 min.^43^ Most of dye molecules were quickly adsorbed to the gemini surfactant/GO composite in the first 20 min, and blue solution appeared slightly faded. As shown in Fig. 7b, the adsorption equilibrium was achieved after ∼60 min for each sample. The colorless transparent solution indicated the efficient removal of dye molecules. The fast equilibrium might be ascribed to the enhanced hydrophobicity of the composites. Some studies on adsorbents modified by surfactants have found that hydrophobic interactions operate during adsorption.^44–47^ Abundant gemini surfactant molecules with two hydrophobic tails existed on the GO surface, resulting in the very fast diffusion of the adsorbate. According to XPS results (Fig. 4c and Table S1†), the nitrogen atom contents of three composites are very close to each other (3.47, 3.38, 3.41 atom% for 10-2-10/GO, 12-2-12/GO, and 14-2-14/GO, respectively), suggesting that their hydrophobic tail chain number or volume is also similar. Thus, the contribution of hydrophobic adsorption in three composites should be similar. Additionally, the hydrophilic adsorption of three composites is similar because of their similar structure. Therefore, the sum of hydrophobic and hydrophilic adsorption, *i.e.*, their total adsorption capacities, are close to each other. The small difference of equilibrium adsorption capacities may be caused by the fact that longer hydrophobic tail chains of surfactants result in stronger hydrophobicity of composites. Thus, the equilibrium adsorption capacity of 14-2-14/GO was slightly higher than those of 10-2-10/GO and 12-2-12/GO.

### 3.3 Adsorption kinetics study

Three kinetic models were adopted to investigate the adsorption process and mechanism. These models included the pseudo-first-order model, pseudo-second-order model, and the Morris–Weber model.

#### 3.3.1 Pseudo-first-order and pseudo-second-order kinetic models

The pseudo-first-order and pseudo-second-order kinetic models are expressed by the following equations, respectively:^48^

2 ln(*Q*_e_ − *Q*_*t*_) = ln *Q* − *k*_1_*t*

3
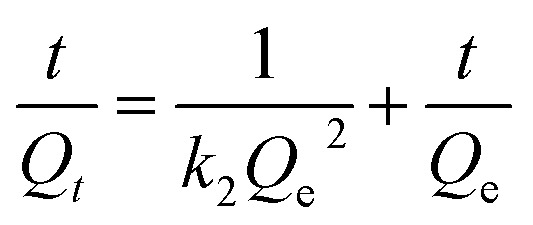
where *Q*_e_ (mg g^−1^) and *Q*_*t*_ (mg g^−1^) are the adsorption capacities at equilibrium and at any time *t* (min), respectively; and *k*_1_ (min^−1^) and *k*_2_ (g mg^−1^ min^−1^) are the rate constants of the pseudo-first-order and pseudo-second-order models, respectively.

**Fig. 8:**
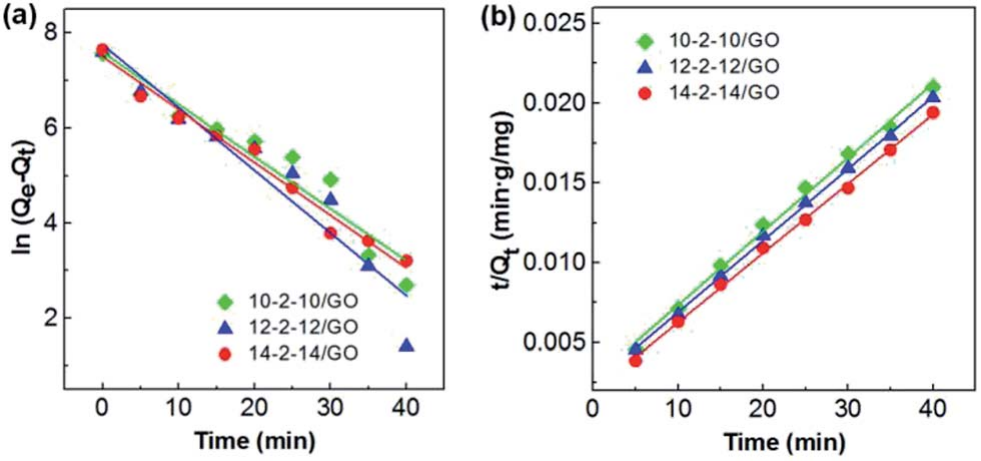
(a) The pseudo-first-order and (b) pseudo-second-order adsorption models for the adsorption of Congo red onto the gemini surfactant/GO composites (adsorbent dose: 5 mg, pH: 3, Congo red concentration: 500 mg L^−1^ and volume: 25 mL).

The pseudo-first-order model and pseudo-second-order model were adopted for predicting experimental procedure in Fig. 8. The kinetic parameters and the correlation coefficients (*r*^2^) are listed in Table S2.†*Q*_e_ calculated by the pseudo-first-order model were very different from the experimental ones. This result suggests that the pseudo-first-order model was unsuited for describing the kinetic for the adsorption of Congo red onto composites. However, the adsorption process fitted well the pseudo-second-order kinetic model, with correlation coefficients higher than 0.99 (Table S2†), and the calculated values of *Q*_e_ were very close to the experimental values. These results indicated the good applicability of the pseudo-second-order model to describe the adsorption of Congo red on the composites.

#### 3.3.2 Morris–Weber model

**Fig. 9:**
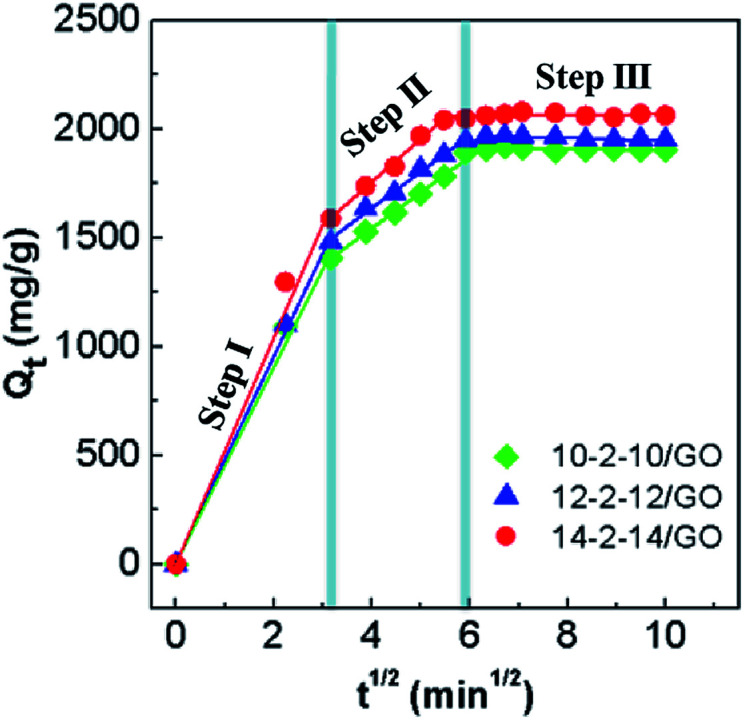
Morris–Weber model model for the adsorption of Congo red on the gemini surfactant/GO composites (adsorbent dose: 5 mg, Congo red concentration: 500 mg L^−1^, and pH: 3).

The actual rate-controlling step was evaluated by the Morris–Weber model in Fig. 9, which can be described as follows:

4 *Q*_*t*_ = *k*_i_*t*^1/2^ + *L*_i_

where *k*_i_ is the rate constant of the Morris–Weber model, *L*_i_ is a constant for any experiment (mg g^−1^), and *Q*_*t*_ has the same meaning as that in eqn (3).

The rate constants and the correlation coefficients (*r*^2^) are listed in Table S3.† Three rate constants of the stepwise adsorption decreased sequentially, *k*_i1_ > *k*_i2_ > *k*_i3_. This result indicated that the adsorption included three diffusion steps. The first steep-sloped period was called as film diffusion in the first 10 min. The external surface adsorption occurred first, and abundant Congo red molecules transferred from the solution to the adsorbent surface. The large slope (*k*_i1_) of the linear portion indicated a very fast diffusion. Then, the second step (between 10 min and 35 min) was controlled by intraparticle diffusion, during which dye molecules migrated slowly from the surface into the internal structure of the adsorbents. The adsorption was almost saturated, leading to increased resistance and a smaller *k*_i2_. The third step was the final equilibrium step (after 35 min), during which dye molecules reached a dynamic balance between the solution, the surface, and internal structure of the composites. The slowest diffusion rate was obtained at this step. The interpretation of this phenomenon had been previously reported.^19,49^

### 3.4 Adsorption isotherm study

Adsorption isotherms were studied by adjusting the initial concentrations of Congo red solutions (100–1000 mg L^−1^). The equilibrium time was based on the previous kinetic study (60 min). The adsorption isotherm data of Congo red onto composites at 298 K was fitted with two classic isotherm models, namely, Langmuir [eqn (5)] and Freundlich [eqn (6)], which are expressed by the following equation:5
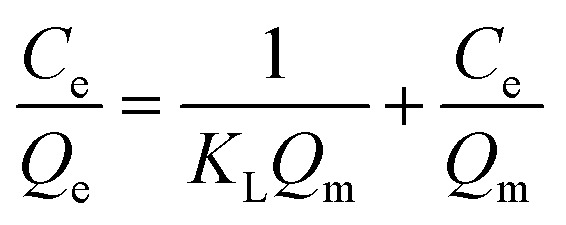
6
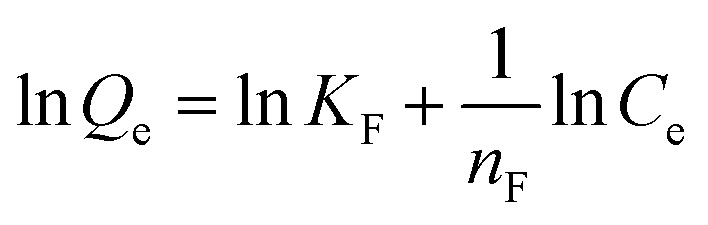
where *Q*_e_ is the adsorption capacity at equilibrium time (mg g^−1^); *C*_e_ is the equilibrium concentration of Congo red (mg L^−1^); *Q*_m_ is the maximal adsorption capacity of the composites (mg g^−1^); *K*_L_ and *K*_F_ are the Langmuir and Freundlich constants, respectively; and *n*_F_ is the influence coefficient of the equilibrium adsorbed amount to solution concentration.

**Fig. 10:**
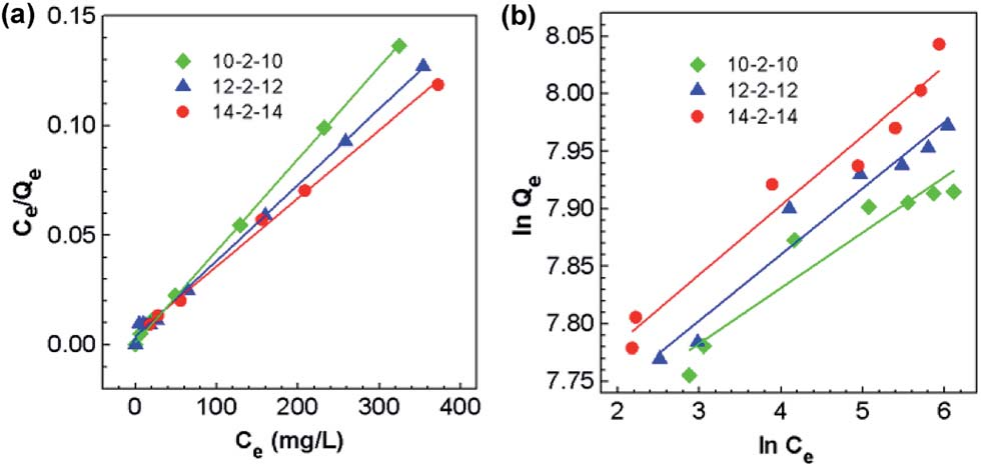
Adsorption isotherms of the adsorption of Congo red onto the gemini surfactant/GO composites: fitting curves of the (a) Langmuir and (b) Freundlich models (*t*: 60 min, pH: 3).

The adsorption isotherms for Congo red onto the gemini surfactant/GO composites are shown in Fig. 10, and the corresponding parameters are listed in Table S4.† The values of the correlation coefficient for the Langmuir model were higher than 0.99, whereas those for the Freundlich model were smaller than 0.98. These results indicated that the monolayer Langmuir adsorption isotherm was more suitable to explain the adsorption instead of the Freundlich model. The gemini surfactant/GO composites would be effective adsorbents for Congo red because of the large values of *Q*_m_ (2116, 2193, and 2325 mg g^−1^ for 10-2-10/GO, 12-2-12/GO and 14-2-14/GO, respectively).

The ultrahigh adsorption capacities of the gemini surfactant/GO composites offer great advantages for the high efficiency removal of Congo red. Table 1 lists a comparison of the results from our work and those of various adsorbents previously reported for the treatment of Congo red. Aluminium oxide is extensively studied because of its fast equilibrium time and high adsorption capacity (*e.g.*, 2 min for 417 mg g^−1^).^50^ Furthermore, the Congo red adsorption capacities of other metal oxides range from 1204 to 2409 mg g^−1^, which is ascribed to their chemical composition and microstructure.^51–53^ Among these adsorbents, both hydrophobic and hydrophilic adsorption contribute to the adsorption of Congo red. Adsorption capacities of the prepared gemini surfactant/GO composites in this study are significantly higher than those of most adsorbents, and their fast adsorption rate also shows great superiority. Therefore, the gemini surfactant/GO composites may be suitable as efficient adsorption materials for the treatment of organic wastewater.

### 3.5 Proposed adsorption mechanism

**Fig. 11:**
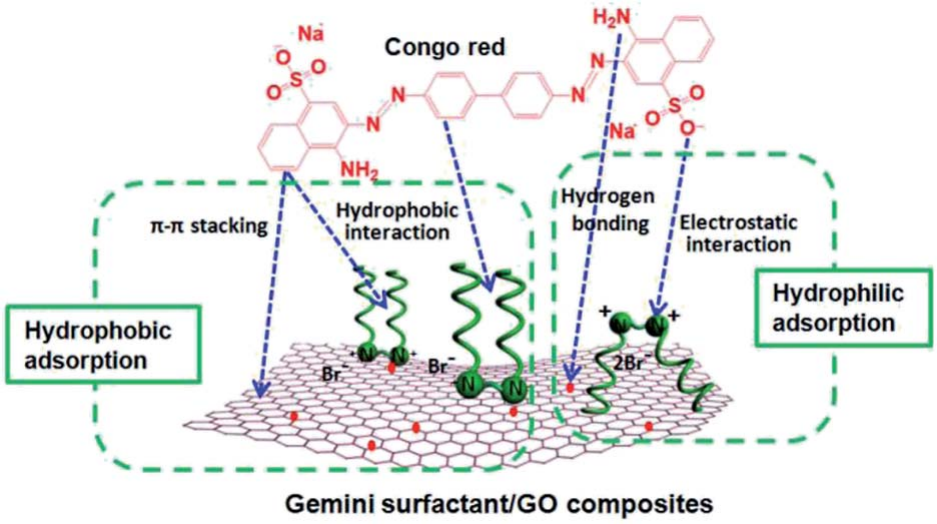
Proposed mechanisms for the adsorption of Congo red on gemini surfactant/GO composites.

In this study, the composites are composed of GO and gemini surfactants. According to previous literatures and the unique structure of the composites in this study, we propose that both hydrophobic and hydrophilic adsorption occur between composites and Congo red (Fig. 11). On the one hand, the hexagonal arrays of the carbon atoms in GO and the hydrocarbon chains of surfactants can supply the hydrophobicity for the composites. Previous studies have reported that π–π stacking interaction exists between the aromatic structure of Congo red molecules and graphene.^21,23^ At the same time, hydrocarbon chains of surfactants could produce hydrophobic interaction with Congo red in adsorption process. On the other hand, the oxygen-containing groups (such as, –COOH, –OH, and –O–) of GO and quaternary ammonium of cationic surfactants provide the hydrophilicity of the composites. Cationic surfactants can produce strong electrostatic adsorption on anionic dyes (such as Congo red).^55^ This process leads to the ultrahigh adsorption capacities of Congo red molecules onto the composites. Moreover, hydrogen bonding may occur between the oxygen-containing groups of graphene and Congo red.^54^ Therefore, the excellent adsorption properties of gemini surfactant/GO composites can be attributed to the synergistic effect of the hydrophobic and hydrophilic interactions between adsorbents and Congo red.

### 3.6 Recyclability of the gemini surfactant/GO composites

**Fig. 12:**
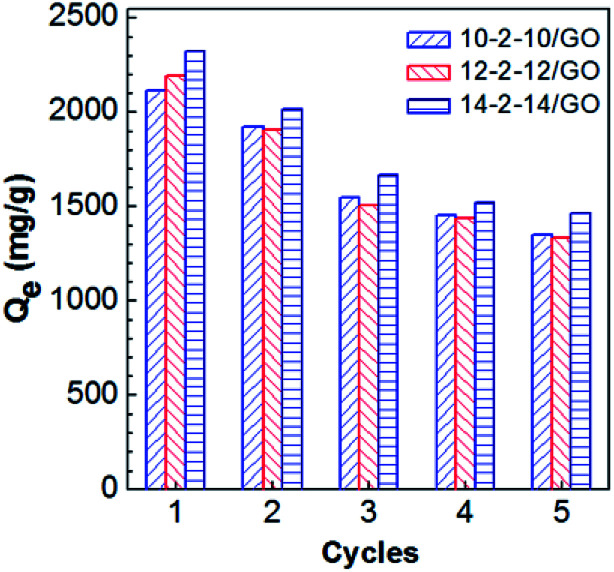
Effect of reuse cycles on *Q*_e_ of gemini surfactant/GO composites.

Recyclability is a key factor of adsorbents for the wastewater treatment.^41^ In this work, the adsorption of Congo red on the composites was investigated for five adsorption–desorption cycles. As shown in Fig. 12, the recycled adsorbents were inferred to retain most of their adsorption abilities in the cycle experiments. The adsorption capacities presented some reduction, because a portion of Congo red adsorbed on composites very tightly and failed to desorb. In addition, wastage of adsorbents in the recovery process contributed to the loss in adsorption capacity. Despite the adsorption capacities of the recycled materials gradually declined, the adsorption capacities after five cycles still remained at 1350, 1336, and 1464 mg g^−1^ for 10-2-10/GO, 12-2-12/GO, and 14-2-14/GO, respectively. Thus, the excellent recovery capabilities of the gemini surfactant/GO composites are demonstrated.

## 4. Conclusions

A series of gemini surfactant-modified GO composites (10-2-10/GO, 12-2-12/GO and 14-2-14/GO) were prepared by a simple mixing process, in which three gemini surfactants with different tail chain lengths were used. Characterization of the composites by SEM, FT-IR, XRD, and XPS confirmed that gemini surfactants were successfully integrated into the composites. The formation of the composites resulted from the hydrophobic binding and electrostatic interactions between the gemini surfactants and GO. The adsorption data proved that the obtained gemini surfactant/GO composites are outstanding absorbents for the removal of Congo red, with maximum absorption capacities of 2116, 2193, and 2325 mg g^−1^ for 10-2-10/GO, 12-2-12/GO and 14-2-14/GO, respectively. The removal process was favorable at acidic pH and reached equilibrium in ∼60 min. The adsorption of Congo red on composites well fitted the Langmuir model of adsorption isotherms and the pseudo-second-order model of kinetics. Finally, the gemini surfactant/GO composites showed excellent recovery capabilities, and the adsorption capacities were more than 1000 mg g^−1^ even at the fifth cycle. The gemini surfactant/GO composites may be promising adsorbents for the treatment of dye-containing wastewater because of their superior adsorption performance.

## Conflicts of interest

There are no conflicts to declare.

## Supplementary Material

RA-009-C8RA10025J-s001
