# Supplementary material for: Preparation of gemini surfactant/graphene oxide composites and their superior performance for Congo red adsorption
Source: RSC Adv. 2019 Feb 8;9(9):4908–16. doi: 10.1039/c8ra10025j (PMC9060756; doi:10.1039/c8ra10025j)
Supplement: RA-009-C8RA10025J-s001 [file RA-009-C8RA10025J-s001.pdf]

Electronic Supplementary Material (ESI) for RSC Advances.
This journal is © The Royal Society of Chemistry 2019

Electronic Supplementary Information

Preparation of gemini surfactant/graphene oxide composites and their superior performance for Congo red adsorption

Shuai He,*[,a] Xingli Liu,[a] Ping Yan,[a] Anqi Wang,[a] Jinzhu Su[a] and Xin Su*[,b]

[a.] *College of Chemistry and Environmental Protection Engineering, Southwest Minzu University, Chengdu 610041, China. E-mail: heshuaifish@126.com; Tel: +86-028-85928021.*

[b.] *Polymer Research Institute, State Key Laboratory of Polymer Materials Engineering, Sichuan University, Chengdu 610065, China. E-mail: xinsu@scu.edu.cn; Tel: +86-028-85461786.*

Table S1 Atomic percentages and Br/N ratios of the gemini surfactant/GO composites obtained by XPS.

Name	Atomic mol%		
	10-2-12/GO	12-2-12/GO	14-2-14/GO
C1s	81.75	82.45	82.81
O1s	12.93	11.79	11.63
N1s	3.47	3.38	3.41
Br3d	1.85	2.38	2.15
Br/N	0.53	0.70	0.63

As shown in Fig. S1, GO exhibits a maximum absorption peak at 233 nm (the π–π* transition of the aromatic C=C bonds) and a shoulder peak at 290-300 nm (the n–π* transition of the C=O bonds). 12-2-12 shows a peak at 190-218 nm. However, these absorption peaks do not appear in the immersion solution of 12-2-12/GO composite. Moreover, after immersion for 100 min, no significant change in the spectra of the immersion solution is observed. These results indicate that the composites possess good stability in water.

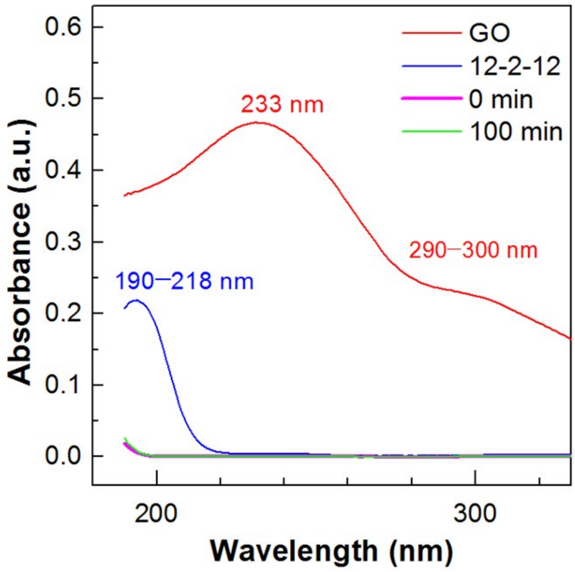

Fig. S1 UV–vis absorption spectra of GO, 12-2-12, immersion solution of 12-2-12/GO composite for 0 min and 100 min.

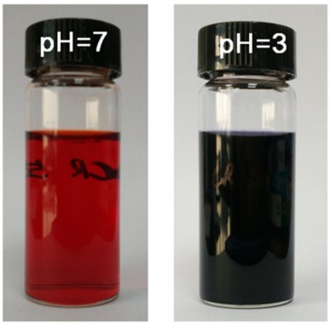

Fig. S2 Photographs of 500 mg/L Congo red solution at pH 7 and 3.

Table S2 Parameters of pseudo-first-order and pseudo-second-order models for the adsorption of Congo red by the gemini surfactant/GO composites.

Gemini surfactant/GO composites	Pseudo-first-order			Pseudo-second-order			Q_e (exp) (mg/g)
	Q_e (mg/g)	k_1 (1/min)	r^2	Q_e (mg/g)	k_2 (g/mg·min)	r^2	
10-2-10/GO	1967	0.1096	0.9181	1950	8.129×10^{-5}	0.9954	1905
12-2-12/GO	2364	0.1332	0.9056	1962	8.473×10^{-5}	0.9990	1949
14-2-14/GO	1707	0.1094	0.9774	2098	9.907×10^{-5}	0.9983	2063

Table S3 Parameters of Morris-Weber model for the adsorption of Congo red on the composites.

Gemini surfactant/ GO composites	Step I		Step II		Step III	
	k_{i1} (mg/g·min$^{1/2}$)	r^2	k_{i2} (mg/g·min$^{1/2}$)	r^2	k_{i3} (mg/g·min$^{1/2}$)	r^2
10-2-10/GO	452.6	0.9880	164.7	0.9904	-2.702	0.5542
12-2-12/GO	473.0	0.9970	166.9	0.9923	-5.750	0.7885
14-2-14/GO	516.6	0.9732	201.4	0.9853	2.756	0.6582

Table S4 Parameters of the Langmuir and Freundlich isotherms for the adsorption of Congo red onto the composites.

Gemini surfactant/GO composites	Langmuir			Freundlich		
	Q_m (mg/g)	K_L (L/mg)	r^2	K_F [(mg/g)(L/mg)$^{1/n}$]	n_F	r^2
10-2-10/GO	2116	0.3675	0.9997	2075	20.70	0.9550
12-2-12/GO	2193	0.08987	0.9987	2059	17.36	0.9767
14-2-14/GO	2325	0.06313	0.9967	2126	16.58	0.9796
